# Prevalence and Associated Factors of Suicidal Ideation and Attempt among Prisoners in Ethiopia: A cross-sectional study

**DOI:** 10.4314/ejhs.v31i3.20

**Published:** 2021-05

**Authors:** Temesgen Tadesse, Endashaw Habtamu, Zehara Reshid, Endalamaw Salelew

**Affiliations:** 1 Department of Psychiatry, College of Health and Medical Science Haremaya University; 2 Department of Psychiatry, College of Health Science Dilla University; 3 Amanuel Mental Specialized Hospital; 4 Department of Psychiatry, College of Medicine and Health Science University of Gondar

**Keywords:** Prisoners, suicidal ideation, suicide attempt, Ethiopia

## Abstract

**Background:**

Suicide is a serious cause of mortality worldwide and the single most common cause of death in the prison population. Studies on suicidal ideation and attempt among prison people in Ethiopia are limited. Therefore, the objective of this study was to assess the prevalence and associated factors of suicidal ideation and attempt among prisoners in Debre Markos Correctional Center, northwest, Ethiopia, 2019.

**Method:**

An institution-based cross-sectional study was conducted using the simple random sampling technique; a total of 640 participants was recruited. Suicidal ideation and attempts were assessed using the suicidality module of the World Mental Health (WMH) survey initiative version of the World Health Organization (WHO) Composite International Diagnostic Interview (CIDI).

**Results:**

The prevalence of suicidal ideation and attempt were 21.9% and 13.1% with 95% CI (18.40, 25.20) and (10.60, 15.80) respectively. The study revealed that female sex (AOR=2.60, 95%CI: 1.39, 8.20) and family history of mental illness (AOR=2.08, 95%CI: 1.11, 3.90) were significantly associated with both suicidal ideation and attempt. Whereas divorced/widowed (AOR=3.67, 95%CI: 2.05, 6.58), common mental disorder (AOR=1.98, 95%CI: 1.25, 3.16) and poor social support (AOR=2.68, 95%CI: 1.42, 5.06) were statistically associated with suicidal ideation, and previous incarceration (AOR=2.38, 95%CI: 1.20, 5.16) was significantly associated with a suicide attempt.

**Conclusion:**

The result shows that the prevalence of suicidal ideation and attempt were serious problems among prisoners. As a result, inmate people need greater attention and interventions for suicidal behaviors.

## Introduction

Suicidal ideation is any self-reported thoughts for killing oneself and an important phase in the suicidal process(1, 2), whereas suicide attempt is a self-injurious behavior with a non-fatal outcome(3). Prisoners are special populations that are under court control with limited, liberty, autonomy, and communication with family and friends(4). An estimated 10.1 million people are in correction institutions worldwide; the majority of them live in low and middle-income countries (LMIC)(5). According to world prison population list report 2014, in Ethiopia 113,727 peoples are found in prison(6). Prisoners had poor general health of both physical and mental illnesses(5). These group of populations are vulnerable with an increase of a nine fold of suicidal risk and a two-fold of suicide rate when compared to the general population(7). A recent study indicates that characteristics of the prison setting itself may increase the risk of suicide(8). Community-based studies reported that the prevalence of suicidal ideation for lifetime and 12 months ranges 10 to14% and 2.3 to 14.6% respectively(9). Suicide is the 10^th^ leading cause of death worldwide and the second leading cause of death among those aged 15–29 years(10). National cost of suicides and suicide attempts in the United States in 2013 was 58.4 billion dollar(11). More than 75% of global suicides occurred in low- and middle-income countries(12). Suicidal behavior is the most common cause of death in correction institutions, and also it is an international problem on prisoners(13). Globally, the rate of suicide found in the range 16–19 deaths per 100,000 inmates each year(14). A meta-analysis conducted in United Kingdom among prisoners found that the risk of suicide was 15 times higher among those who experienced recent suicidal ideation(13). Suicide is the third leading cause of death in US correctional institutions and the second in jails(15). Studies carried out on suicidal ideations and attempt among prisoners across the globe reported that, the prevalence of suicidal ideation and attempt ranges 8.04%–70%(16, 17), and 9.3%–38.9% respectively(18, 19), and risk factors were sex, young age, unmarried, low education, family history of suicide and mental illness, perceived stigma, lack of support, duration of stay, comorbid mental disorders and substance use problems(7, 8, 13, 16, 20–23). Besides, the burdens and consequences of the problems, suicidal ideation and attempt among these groups of people have not been well explored in Ethiopia. As a result, studies on this area could provide essential data for the government and correctional institutions to alert for early detection and interventions of the problems. Therefore, the objective of this study was to determine the prevalence and associated factors of suicidal ideation and attempt among prisoners in the Debre Markose correctional center, northwest, Ethiopia, from May to June, 2019.

## Methods and Materials

**Study setting and population:** An institution-based cross-sectional study was carried out from May to June/2019. The study was conducted at the Debre Markos correction center, found in Amhara region, 299km from Addis Ababa the capital of Ethiopian (24). The correction center had 1503 sentenced prisoners for different types of crimes. All prisoners aged 18 years and above in the correction center were source population, whereas all sampled sentenced prisoners were study populations after excluding those severely ill and/or inability to communicate at the moment.

**Sample size determination and technique**

The sample size was determined using t h e single population proportion formula by taking the prevalence of suicidal ideation 16.6% and attempt 9.3% a study reported at the Jimma correction center southwest Ethiopia(18). Thus, to obtain the maximum sample size, the prevalence of suicidal ideation was considered with; 3%, margin of error, 95% confidence level. After adding 10% of non-response rate, the total sample size was 650. Then, computer generated simple random sampling technique was used to select study participants.

**Data collection tools and procedures**

Data were collected using a pretested interviewer administered Amharic versions of the tool for a month. The questionnaire was designed in English and translated to Amharic, and back to English to maintain consistency. Data collectors were trained on how to interview participants and explained about ethical principles, such as aim of the study, confidentiality, data management and securing respondents' informed consent for participation. The measurement had socio-demographic, clinical, substance use, prison related, social support, perceived stigma, common mental disorders' characteristics and suicidal ideation and attempt.

**Social support** was measured using Oslo 3-item social support scale with scores ranging from 3–14: 3–8 = poor support, 9–11 = moderate support and 12–14 = strong support(25).

**Perceived stigma** was assessed by Jacoby 3-item perceived stigma scale with a score of one and above were considered as having perceived stigma(26).

**Common Mental disorders** were assessed using self-reporting questionnaire (SRQ) screening instrument had 20 yes/no questions on depression, anxiety, and somatic symptoms experienced in the last 30 days. This was developed by the World Health Organization (WHO) and has been tested in numerous settings and with a cut-off point 6 above was found to have a specificity of 83.7% and sensitivity 84.8%(27).

**Suicidal ideation and attempt** were measured with the suicidality module of World Mental Health (WMH) survey initiative version 3.0 of the World Health Organization (WHO) composite International diagnostic interview (CIDI) validated in the country and its internal consistency and percentage of agreement was 0.78, and 93.2%–100% for full scale respectively(28). The respondent who answers yes, to the question “Have you ever seriously thought about committing suicide?” were considered as having suicidal ideation, and the respondents who answer yes, to the question “Have you ever attempted suicide?” were considered as have suicide attempt. In this study, its internal consistency was in a good range (Cronbach's alpha α=0.89).

**Data processing and analysis**: The collected data were coded, checked for completeness, consistency and entered into EPI-data version 3.1 and then exported to the Statistical Package for Social Science (SPSS) version 20 for analysis. Descriptive statistics were computed and presented using frequencies, percentages and tables. Binary logistic regression analysis was used to determine factors associated with suicidal ideation and attempt. In the bivariate logistic regression analysis, variables found to have p-values < 0.25 were candidates for the multivariate logistic regression analysis. In the multivariate analysis, variables with a p-value < 0.05 were considered significantly associated with the outcome variable. The strength of the associations was explained with by odds ratio (OR) at 95% confidence interval(CI).

**Ethical consideration:** Ethical clearance was obtained from the ethical review committee (ERC) of the College of Medicine and Health Science of the University of Gondar. Letter of permission was obtained from the Amanuel Mental Specialized Hospital and submitted to the Debre Markos correctional center. The participants were provided sufficient information about the purpose, objectives and relevance of the study, and allowed them to decide about the right to participate or refuse. Informed written consent was received from the study participants. Confidentiality was maintained by omitting personal identifiers. This study was conducted in accordance with the Declaration of Helsinki.

## Results

**Socio-demographic characteristics:** A total of 650 participants was invited in the study with a response rate of 98.5%. The median age of the study participants was 28 years with interquartile range (IQR) of 11 years. The majority, 287 (44.8%), were age groups of 18–27 years, 578 (90.3%) were male, 418 (65.3%) were urban residency, 635 (99.2%) were Amhara, 623 (97.3%) were Orthodox and 326(50.9%) were single in marital status. Some participants, 286 (44.7%), were primary school education, 245 (38.3%) were farmers, 219 (34.2%) had done theft and robbery of crime followed by 202 (31.6%) murder (Table 1).

**Table 1:** Socio-demographic characteristic distribution of study participants(n=640)

Variable	Categories	Frequency	Percentage
Age	18–27	287	44.8
	28–37	223	34.8
	38–47	92	14.4
	>47	38	6.0
Sex	Male	578	90.3
	Female	62	9.7
Residency	Urban	418	65.3
	Rural	222	34.7
Religion	Orthodox	623	97.3
	Muslim	11	1.7
	Protestant	6	0.9
Marital Status	Single	326	50.9
	Married	216	33.8
	Divorced/Widowed	98	15.3
Ethnicity	Amhara	635	99.2
	Other *	5	0.8
Educational Level	Unable to write & read	137	21.4
	Primary school	286	44.7
	High school	149	23.3
	Diploma and above	68	10.6
Occupation	Employed	79	12.3
	Trading	65	10.2
	Farmer	245	38.3
	Student	53	8.3
	Unemployed	172	26.9
	Other**	26	4.0
Type of Crime	Theft & robbery	219	34.2
	Murder	202	31.6
	Rape	35	5.5
	Corruption	30	4.7
	Physical attack	128	20.0
	Emotional attack and cheating	26	4.0

NB.

* includes ethnic groups (Tigray, Oromo)

** includes occupations (House wife, Daily laborer and driver)

**Clinical related factors:** Of the respondents, 390 (60.9%) had common mental disorders, 36 (5.6%) and 23 (3.6%) had a family history of suicide attempt and committed suicide respectively. Some, 78(12.2%), had a family history of mental illness, 75(11.7%) and 82 (12.8%) had a history of mental and chronic physical illnesses respectively (Table 2).

**Table 2:** Clinical related characteristics of the study participants (n=640)

Variables	Categories	Frequency	Percentage
Family history of suicide attempt	Yes	36	5.6
	No	604	94.4
Family history of suicide commits	Yes	23	3.6
	No	617	96.4
Family history of mental illness	Yes	78	12.2
	No	562	87.8
History of mental illness	Yes	75	11.7
	No	565	88.3
Common mental disorder	Yes	390	60.9
	No	250	39.1
Chronic physical illness	Yes	82	12.8
	No	55	87.2
Types of chronic physical illness	Hypertension	31	37.8
	Diabetes mellitus	22	26.8
	Cardiac disease	15	18.3
	
	Others***	14	17.1

*** **Others**: HIV/AIDS, Asthma, Leprosy

**Social support and perceived stigma**: Out of the participants, 298 (46.6%), 222 (34.7%) and 120 (18.7%) had poor, moderate and strong social support respectively. The majority, 355 (55.5%) of the respondents had perceived stigma based on the Jacoby perceived stigma scale with cutoff point one and above, whereas 285 (45.5%) have no perceived stigma.

**Magnitude of suicidal ideations and attempts**: The lifetime prevalence of suicidal ideations and attempts was 140 (21.9%) and 84 (13.1%) with 95% CI (18.40, 25.20) and (10.60, 15.80) respectively. In the last one month 68 (48.6%) had thought about committing suicide and 20(23.8%) had suicide attempts. Of the participants, 60 (42.9%) had planned to commit suicide in their lifetime. The major reasons for suicide attempts were hopelessness due to crime 35 (41.7%) and feel guilty of crime committed 24 (28.6%) (Table 3).

**Table 3:** prevalence of suicidal ideations and attempts among the study participants (n=640)

Variables	Category	Frequency	Percentage
Ever suicidal ideation	Yes	140	21.9
	No	500	78.1
Suicidal ideation in the last one month	Yes	68	48.6
	No	72	51.4
Ever plan of suicide	Yes	60	42.9
	No	80	57.1
Ever suicide attempt	Yes	84	13.1
	No	556	86.9
Suicide attempt in 1 month	Yes	20	23.8
	No	64	76.2
Frequency of suicide attempt	Once	56	66.7
	Twice	18	21.4
	Three or more	10	11.9
Reason for suicide attempt	Hopelessness due to crime	35	41.6
	Feel guilty of crime committed	24	28.6
	Family conflict	15	17.9
	Economic problem/poverty	10	11.9
Severity related to attempt	Seriously attempted	47	56.0
	Methods used not effectively	29	34.5
	To seek help	8	9.5
Methods of attempt	Hanging	35	41.7
	Poisoning	24	28.6
	Use sharp materials	15	17.8
	Jump from a high place and electricity	10	11.9

**Factors associated with suicidal ideations**: In the bivariate logistic regression analysis, sex, marital status, family history of suicide attempt and suicide commit, family history of mental illness, common mental disorder, ever tobacco use poor social support and perceived stigma were found to be a candidate for multivariate analysis at p-value < 0.25. In the multivariate logistic regression analysis female sex, divorced/widowed, family history of mental illness, common mental disorder and poor social support were statistically significant with suicidal ideation at p-value less than 0.05.

The study showed that female sex was 2.60 times more likely to develop suicidal ideation as compared to male sex (AOR=2.60, 95%CI: 1.39, 8.20). Divorced/ widowed were 3.67 times more likely to develop suicidal ideation compared to married (AOR=3.67, 95%CI: 2.05, 6.58). Family histories of mental illness were 2.49 times higher risk compared to no family history of mental illness (AOR=2.49, 95%CI: 1.41, 4.38). Having poor social support were more than two times more likely to result in suicidal ideation compared to strong social support (AOR=2.68, 95%CI: 1.42, 5.06). Common mental disorders were 1.98 times more likely to lead to suicidal ideation than who had not common mental disorder (AOR=1.98, 95%CI: 1.25, 3.16) (Table 4).

**Table 4:** Bivariate and multivariate logistic regression analysis of suicidal ideation and associated factors among study participants (n=640)

Explanatory variables	Categories	Suicidal ideations		COR, (95% CI)	AOR (95% CI)
		Yes	No		
**Sex**	Female	18	44	3.53(1.49,6.30)	2.60(1.39, 8.20)**
	Male	122	456	1	1
**Marital status**	Married	37	179	1	1
	Single	56	270	1.00(0.64, 1.58)	1.17(0.72, 1.92)
	Divorced/widowed	47	51	4.46(2.62,7.59)	3.67(2.05, 6.58)**
**Family history of**	Yes	17	19	3.50(0.1.77, 6.93)	1.23(0.97, 4.13)
**suicide attempt**	No	123	481	1	1
**Family history of**	Yes	9	14	2.38(1.01, 5.63)	2.54(0.56, 6.69)
**suicide commits**	No	131	486	1	1
**Family history of**	Yes	31	47	2.74(1.66,4.52)	2.49(1.41, 4.38)**
**mental illness**	No	109	453	1	1
**Perceived stigma**	Yes	87	268	1.42(0.97, 2.09)	1.34,(0.88, 2.05)
	No	53	232	1	1
**Social support**	Poor	83	215	2.34(1.32, 4.15)	2.68(1.42,5.06)**
	Moderate	40	182	1.33(0.72, 2.47)	1.35(0.69, 2.65)
	Strong	17	103	1	1
**Common mental**	Yes	102	288	1.98(1.31, 2.99)	1.98(1.25, 3.16)**
**disorder**	No	38	212	1	1
**Ever tobacco use**	Yes	23	43	2.09(1.21, 3.61)	1.72(0.39,3.29)
	No	117	457	1	1

**N.B.** 1 references, significances: **p-value less than 0.01; Hosmer lemeshow test = 0.626, Chi square=6.19, df=8

**Factors associated with suicide attempt**: In the bivariate logistic regression analysis sex, marital status, family history of suicide attempt and suicide commit, family history of mental illness, ever alcohol use, ever tobacco use and previous incarceration were associated with suicidal attempts at p-value less than 0.25. In the multivariate logistic regression analysis variables; female sex, family history of mental illness and previous incarceration were found to be statistically associated at p-value < 0.05. The study revealed that the female sex was 2.14 times more likely to have suicidal attempt compared to male sex (AOR=2.14, 95%CI: 1.89, 9.40) and family history of mental illness was 2.08 times higher risk compared to not having a family history of mental illness (AOR=2.08, 95%CI: 1.10, 3.90).

On the other hand, previous incarcerations were 2.38 times more likely to lead to suicide attempt compared to no previous incarcerations (AOR=2.38, 95%CI: 1.20, 5.16) (Table 5).

**Table 5:** Bivariate and multivariate logistic regression analysis of suicide attempt and associated factors among study participants (n=640)

Explanatory variables	Categories	Suicide attempt		COR, (95% CI)	AOR (95% CI)
		Yes	No		
**Sex**	Female	15	47	2.35(2.03,10.7)	2.14(1.89,9.40)**
	Male	69	509	1	1
**Marital status**	Married	24	192	1	1
	Single	38	288	1.06(0.61,1.82)	1.07(0.6,1.89)
	Divorced/widowed	22	76	2.32(1.23,4.38)	1.9(0.87,3.72)
**Family history of** **suicide attempt**	Yes	10	26	2.75(1.28,5.94)	2.26(0.76,5.76)
	No	74	530	1	1
**Family history of** **suicide commits**	Yes		16	1.95(1.12,4.74)	1.28(0.78,2.71)
	No	77	540	1	1
**Family history of** **mental illness**	Yes	19	59	2.46(1.38,4.39)	2.08(1.10,3.90)*
	No	65	497	1	1
**Ever alcohol use**	Yes	54	300	1.48(0.95,2.47)	1.58(0.65,2.62)
	No	31	255	1	1
**Previous** **incarceration**	Yes	11	35	2.24(1.09,4.61)	2.38(1.20,5.16)*
	No	73	521	1	1
**Ever tobacco use**	Yes	15	51	2.15(1.15,4.04)	1.66(0.82,3.34)
	No	69	505	1	1

**NB.** 1 references, significances:*p-value < 0.05 **p-value < 0.01; Hosmer lemeshow test = 0.727, Chi square=3.626, df= 6,

## Discussion

This study examined the magnitude and associated factors of suicidal ideation and attempt among prisoners, revealed a prevalence of 21.9% and 13.1% respectively. The prevalence of suicidal ideation is consistent with a study carried out on male prisoners in Pakistan 22% (29).

The prevalence of suicidal ideation in the current study was higher than those of two different studies in Ethiopia 16.6%(18), and 8.04%(16), and 16.9% in Russia (30), 16% in USA(31), and 14.9% in Colombia (22). The possible reason for the discrepancies might be variations in study populations. For instance, only male inmates who had less chance to develop suicidal behaviors were sampled in Russia. Besides, the differences in findings also relate to the tools used. For example, in Russia the 4-item suicidality scale from Affective Disorders and Schizophrenia Present and Lifetime Version (ADS-PL) was used, a 12-item suicidality scale from the Personality Assessment Inventory (PAI) was put to use in USA, whereas the suicide orientation inventory tool questionnaire was utilized in Colombia. Moreover, the outcomes might differ due to the socio-demographic factors.

On the contrary, the prevalence of suicide ideation among inmates is lower than that of studies done in Belgium 43.1%(32), Australia 34%(14), Italy 43.7%(33), China 70%(17) and Iran 44.6%(19). The possible reasons for the differences might be tools. For example, paykel suicidal scale (PSS), Mini International Neuropsychiatric Interview (MINI), Suicide Ideation scales (SSI); Symptoms Check List-90-Revised (SCL-90-R) were used in Belgium, Italy, China and Iran respectively. As a matter of fact, sociocultural factors also responsible for the variations of the results.

On the other hand, this study showed that the prevalence of suicide attempt was higher than a study carried out in Ethiopia 9.3%(18). The possible reason for variations might be differences in tool and sample size. However, this result was lower than those of studies conducted in Belgium 20.3% (32), Australia 21%(14) and Iran 38.9%(19). The reason for variations might be the sampling technique used. For instance, in Australia stratified sampling with telephone survey this is not easy to record what the participants said. Besides, the differences of the tools used and sociodemographic factors also reasons for the outcomes.

According to our study, women were significantly associated with suicidal ideation and attempt than their counterparts. Our finding was supported by studies in England, Israel and India(34, 35). This might be the differences in biological make up, socio-cultural influences on the women to express their problems than, and the suppressed emotion might result in suicidal behaviors can lead to develop such behaviors. But, inconsistent with a study in Ethiopia reported that male sex was significantly associated with suicidal ideation(16). This needs further study.

Prisoners who had family history mental illness were significantly associated with suicidal ideation and attempt. This is supported by a study carried out in Israel(35). According to our finding, divorced/widowed prisoners were 3.67 times more likely to develop suicidal ideation compared to married. This is consistent with a study conducted in Chicago (21). The inmates who had poor social support were 2.68 times more likely to result in suicidal ideation compared to strong social support. This is supported by other studies conducted in Belgium(32) and in North Russia(30). This might be the fact that minimal friends inside or outside correctional facility, weak family support, and limited external contact are known to be major stressors for incarcerated individuals which contributed for suicidal ideation. Furthermore, in this study there is a strong association between common mental disorder and suicidal ideation. This is inline with the study conducted in French(7).

On the other hand, repeated incarcerations were significantly associated with suicide attempt. This is supported by a study conducted in Italy(33), and Ethiopia (18). The possible justifications are the repeated imprisonment may lead to loneliness, loss of a spouse and being deprived of the important resources in the community.

That the study used a standard tool to assess suicidal ideations and attempts is, we feel, its strength. Since, the data were gathered by the interviewer administered method and respondents might reply in favorable of others either over or under reporting. Our work could not rule out recall bias; because the information collected was not being cross-checked. Finally, the cross-sectional study could not allow establishing temporal cause-effect relationships.

In conclusions, the prevalence of suicidal ideations and attempts were serious problems in this study. Female sex and a family history of mental illness were statistically associated with both suicidal ideation and attempt. Whereas, been divorced/ widowed, poor social support and common mental disorder were significantly associated with suicidal ideation. In the other way, prior incarceration was significantly associated with suicide attempt. As a result, inmate people need greater attention and interventions for suicidal behaviors.
